# Integrin beta 1 inhibition alleviates the chronic hyperproliferative dermatitis phenotype of SHARPIN-deficient mice

**DOI:** 10.1371/journal.pone.0186628

**Published:** 2017-10-17

**Authors:** Emilia Peuhu, Siiri I. Salomaa, Nicola De Franceschi, Christopher S. Potter, John P. Sundberg, Jeroen Pouwels

**Affiliations:** 1 Turku Centre for Biotechnology, University of Turku, Turku, Finland; 2 Turku Drug Research Doctoral Programme, University of Turku, Turku, Finland; 3 The Jackson Laboratory, Bar Harbor, Maine, United States of America; 4 TEHO adaptive clinical trial design, University of Helsinki, Helsinki, Finland; University of Bergen, NORWAY

## Abstract

SHARPIN (Shank-Associated RH Domain-Interacting Protein) is a component of the linear ubiquitin chain assembly complex (LUBAC), which enhances TNF-induced NF-κB activity. SHARPIN-deficient (*Sharpin*^*cpdm/cpdm*^) mice display multi-organ inflammation and chronic proliferative dermatitis (cpdm) due to TNF-induced keratinocyte apoptosis. In cells, SHARPIN also inhibits integrins independently of LUBAC, but it has remained enigmatic whether elevated integrin activity levels in the dermis of *Sharpin*^*cpdm/cpdm*^ mice is due to increased integrin activity or is secondary to inflammation. In addition, the functional contribution of increased integrin activation to the *Sharpin*^*cpdm/cpdm*^ phenotype has not been investigated. Here, we find increased integrin activity in keratinocytes from *Tnfr1*^*-/-*^ *Sharpin*^*cpdm/cpdm*^ double knockout mice, which do not display chronic inflammation or proliferative dermatitis, thus suggesting that SHARPIN indeed acts as an integrin inhibitor *in vivo*. In addition, we present evidence for a functional contribution of integrin activity to the *Sharpin*^*cpdm/cpdm*^ skin phenotype. Treatment with an integrin beta 1 function blocking antibody reduced epidermal hyperproliferation and epidermal thickness in *Sharpin*^*cpdm/cpdm*^ mice. Our data indicate that, while TNF-induced cell death triggers the chronic inflammation and proliferative dermatitis, absence of SHARPIN-dependent integrin inhibition exacerbates the epidermal hyperproliferation in *Sharpin*^*cpdm/cpdm*^ mice.

## Introduction

Mice that lack the multifunctional adaptor protein SHARPIN (*Sharpin*^*cpdm/cpdm*^) display progressive multi-organ inflammation, which most prominently manifests with chronic eosinophilic hyperproliferative dermatitis characterized by skin thickening, keratinocyte hyperproliferation and severe inflammation [1,2], which are characteristics of psoriasiform dermatitis. Importantly, these pathological features in the skin are cell autonomous, as *Sharpin*^*cpdm/cpdm*^ grafts on wild type mice maintain the inflammatory phenotype [3]. As an important component of the Linear Ubiquitination Assembly Complex (LUBAC) [4–6], SHARPIN regulates canonical NF-κB (Nuclear factor-kappaB) signaling downstream of several receptors, such as Tumor Necrosis Factor receptor (TNFR) [7], Toll-like receptor 2 [8], Toll-like receptor 3 [9], Interleukin1 receptor [4–6] and CD40 [5,10]. The role of NF-κB in the skin epidermis is controversial as some studies point to a pivotal role for NF-κB signaling in restraining epidermal growth (for example [11–13]), while other studies suggest that NF-κB signaling does not regulate development and differentiation of the epidermis (such as [14,15]). Importantly, crosses between *Sharpin*^*cpdm/cpdm*^ mice and *Tnf*^*-/-*^ (Tnf^tm1Jods^) or *Tnfr1*^*-/-*^ (Tnfrsf1a^tm1Imx^) mice almost fully rescue the inflammatory and dermatitis phenotypes of *Sharpin*^*cpdm/cpdm*^ mice [6,16,17], although other phenotypes were not rescued, such as abnormal leukocyte cell count, the absence of Peyer’s patches and absence of marginal zones in spleen [6,16]. Mechanistically, an increase in TNF-induced keratinocyte apoptosis in the absence of LUBAC-mediated NF-kB signaling drives the systemic inflammation and hyperproliferative dermatitis in *Sharpin*^*cpdm/cpdm*^ mice [6,16,17].

Integrins are the major cell adhesion receptors that mediate the interaction of a cell with the surrounding extracellular matrix, including the basement membrane. Binding of integrins to extracellular ligands triggers a conformational change in integrin structure that allows recruitment of a plethora of cellular factors resulting in activation of several signaling pathways [18]. SHARPIN acts as an integrin inhibitor through binding to the integrin cytoplasmic domain, preventing the recruitment of integrin activating proteins and supporting the integrin inactive conformation [19]. Functionally, SHARPIN-mediated integrin inhibition regulates cell adhesion and migration [19], as well as lymphocyte detachment during transmigration [20]. Importantly, SHARPIN plays mutually exclusive roles in regulating integrins and LUBAC such that SHARPIN inhibits integrins independent of LUBAC [21].

Deregulated integrin activity is implicated in many human pathological conditions, including immune diseases, skin blistering, bleeding disorders, and cancer [18]. In the skin, integrin expression is predominantly confined to the basal keratinocytes that anchor the epidermis to the basal lamina [22,23]. Transgenic mice overexpressing integrin beta 1 (Itgb1) in the suprabasal layer of the epidermis (Tg(Itgb1)0869Fmw) exhibit epidermal hyperproliferation, perturbed keratinocyte differentiation and skin inflammation [24], which resembles the *Sharpin*^*cpdm/cpdm*^ phenotype. Furthermore, integrin blocking therapies have been shown to alleviate psoriasis in mice [25] and in human patients [26]. These data suggest that increased integrin activation in *Sharpin*^*cpdm/cpdm*^ mice may contribute to the dermatitis phenotype. We have previously demonstrated increased Itgb1 activity in the basal layer of the epidermis of *Sharpin*^*cpdm/cpdm*^ mice [19]. However, whether this was due to the absence of SHARPIN-mediated integrin inhibition or secondary to the chronic inflammation-driven proliferative dermatitis has remained unclear.

Using *Tnfr1*^*-/-*^ *Sharpin*^*cpdm/cpdm*^ double knockout mice we now present data suggesting that SHARPIN acts as an integrin inhibitor *in vivo* also in the absence of chronic inflammation. In addition, we demonstrate that Itgb1 inhibition with a function blocking antibody alleviates the excessive proliferation and apoptosis observed in *Sharpin*^*cpdm/cpdm*^ epidermis, but does not ameliorate the chronic and systemic inflammation in *Sharpin*^*cpdm/cpdm*^ mice, suggesting that increased integrin activity in the absence of SHARPIN exacerbates the hyperproliferative skin phenotype in *Sharpin*^*cpdm/cpdm*^ mice.

## Results

### Itgb1 activation is elevated in basal keratinocytes from Tnfr1-/- Sharpincpdm/cpdm mice with normal epidermal thickness

**Fig 1:**
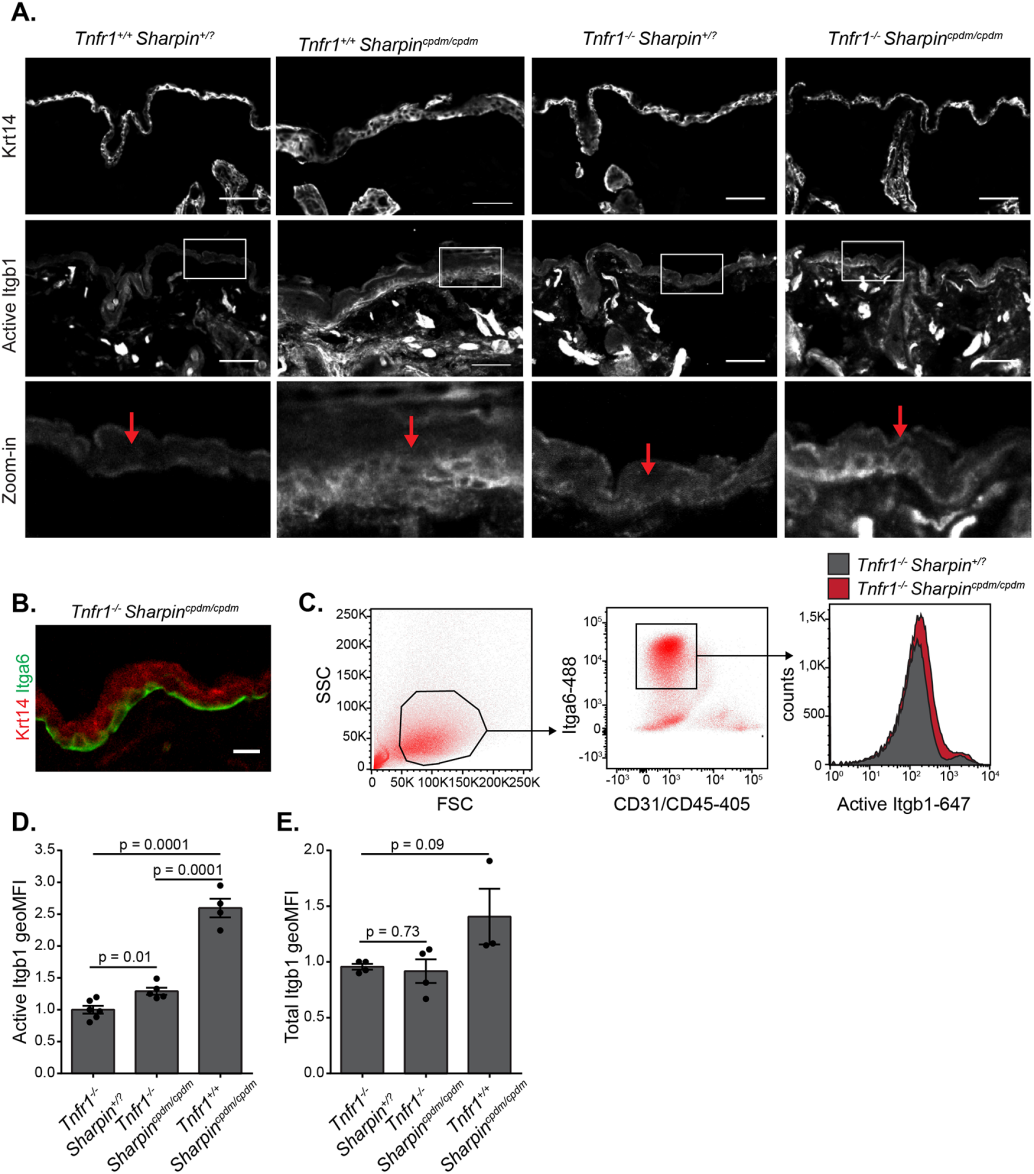
Keratinocytes from *Tnfr1*^*-/-*^ *Sharpin*^*cpdm/cpdm*^ double knockout mice have higher levels of active Itgb1. (A) Representative frozen skin sections from 6 weeks old *Tnfr1*^*+/+*^ *Sharpin*^*+/*?^, *Tnfr1*^*+/+*^ *Sharpin*^*cpdm/cpdm*^, *Tnfr1*^*-/-*^ *Sharpin*^*+/*?^ and *Tnfr1*^*-/-*^ *Sharpin*^*cpdm/cpdm*^ mice (n = 3 animals per genotype) stained for Keratin-14 (Krt14) and active Itgb1 (clone 9EG7). Scale bars represent 50 μm. Arrows indicate the basal cell layer. (B) Representative frozen skin sections from 6 weeks old *Tnfr1*^*-/-*^ *Sharpin*^*cpdm/cpdm*^ mice stained for Krt14 (epidermis) and Itga6 (basal keratinocytes). Scale bars represent 10 μm. (C) Primary keratinocytes were isolated from *Tnfr1*^*-/-*^ *Sharpin*^*+/*?^, *Tnfr1*^*-/-*^ *Sharpin*^*cpdm/cpdm*^ and *Tnfr1*^*+/+*^ *Sharpin*^*cpdm/cpdm*^ mice and studied by flow cytometry. Live cells (FSC/SSC; left panel) were sorted for expression of Itga6 (CD49f) and lineage (Lin) markers CD31 and CD45 (central panel). Cell surface expression of active Itgb1 (CD29; clone 9EG7) was plotted (right panel) from the gated basal keratinocyte population (live, Itga6^+^Lin^-^). (D,E) Relative geometric mean fluorescence intensities of (**D**) active Itgb1 (n = 6, 5 and 4 animals per genotype) and (E) total Itgb1 (n = 4, 4 and 3 animals per genotype) cell surface staining on *Tnfr1*^*-/-*^ *Sharpin*^*+/*?^, *Tnfr1*^*-/-*^ *Sharpin*^*cpdm/cpdm*^ and *Tnfr1*^*+/+*^ *Sharpin*^*cpdm/cpdm*^ basal keratinocytes sorted as in (**C**). Numerical data are mean ± s.e.m.

We previously showed increased levels of active Itgb1 in the basal layer of the epidermis of *Sharpin*^*cpdm/cpdm*^ mouse skin [19], consistent with SHARPIN-mediated integrin inhibition. However, the increase in integrin activation could be secondary to the chronic inflammation-driven proliferative dermatitis in *Sharpin*^*cpdm/cpdm*^ mice. To address whether SHARPIN truly acts as an integrin inhibitor *in vivo*, we made use of the *Tnfr1*^*-/-*^ *Sharpin*^*cpdm/cpdm*^ double knockout mice, in which the skin thickening (Fig 1A), chronic inflammation and dermatitis, typical to *Sharpin*^*cpdm/cpdm*^ mice, are absent [16,17]. This allowed us to investigate the role of SHARPIN *in vivo* without the consequences of increased Tnfr1-induced inflammation and apoptosis in *Sharpin*^*cpdm/cpdm*^ skin.

Labeling skin sections from *Tnfr1*^*+/+*^ *Sharpin*^*+/*?^, *Tnfr1*^*+/+*^ *Sharpin*^*cpdm/cpdm*^, *Tnfr1*^*-/-*^ *Sharpin*^*+/*?^ and *Tnfr1*^*-/-*^ *Sharpin*^*cpdm/cpdm*^ mice with an antibody that specifically recognizes active Itgb1 [9EG7; [27]] confirmed the increased active Itgb1 levels in *Tnfr1*^*+/+*^ *Sharpin*^*cpdm/cpdm*^ epidermis [19] but also revealed elevated Itgb1 activity in *Tnfr1*^*-/-*^ *Sharpin*^*cpdm/cpdm*^ basal keratinocytes despite normal skin thickness (Fig 1A).

To analyze this observation quantitatively and without the interference of high dermal Itgb1 activity (Fig 1A), we isolated keratinocytes from the epidermal layer of *Tnfr1*^*+/+*^ *Sharpin*^*cpdm/cpdm*^, *Tnfr1*^*-/-*^ *Sharpin*^*+/*?^ and *Tnfr1*^*-/-*^ *Sharpin*^*cpdm/cpdm*^ mice, and investigated Itgb1 activity specifically in basal cell layer keratinocytes that also express Integrin alpha 6 (Itga6, also known as CD49f; Fig 1B) (see Methods for details). In order to distinguish basal keratinocytes by flow cytometry, any residual cells expressing lineage markers for leukocytes [CD45, Protein Tyrosine Phosphatase, Receptor Type C (PTPRC)] or endothelial cells [CD31, Platelet And Endothelial Cell Adhesion Molecule 1 (PECAM1)] were gated out by flow cytometry, and cells expressing Itga6 were further analyzed for binding active Itgb1 (9EG7) or total Itgb1 (HMβ1–1) antibodies (Fig 1C). Quantification of these data showed that the amount of active Itgb1 on the surface of the double knockout *Tnfr1*^*-/-*^ *Sharpin*^*cpdm/cpdm*^ basal keratinocytes was indeed significantly increased compared to *Tnfr1*^*-/-*^ *Sharpin*^*+/*?^ keratinocytes, although it was lower than in the hyperproliferative *Tnfr1*^*+/+*^ *Sharpin*^*cpdm/cpdm*^ cells (Fig 1D). The total Itgb1 levels were not significantly altered even though total Itgb1 levels seem elevated in *Tnfr1*^*+/+*^ *Sharpin*^*cpdm/cpdm*^ basal keratinocytes (Fig 1E). Importantly, FACS experiments with primary keratinocytes from four individual mice showed that staining with the 9EG7 antibody results in approximately 6 fold higher signals than isotype control (Panel A and B in S1 Fig). Altogether, these data suggest that increased Itgb1 activity in *Sharpin*^*cpdm/cpdm*^ mice is not solely due to chronic inflammation and proliferative dermatitis but that SHARPIN functions as an integrin inhibitor *in vivo*.

### Itgb1 inhibition ameliorates the epidermal hyperproliferation phenotype in *Sharpin*^*cpdm/cpdm*^ mice

**Fig 2:**
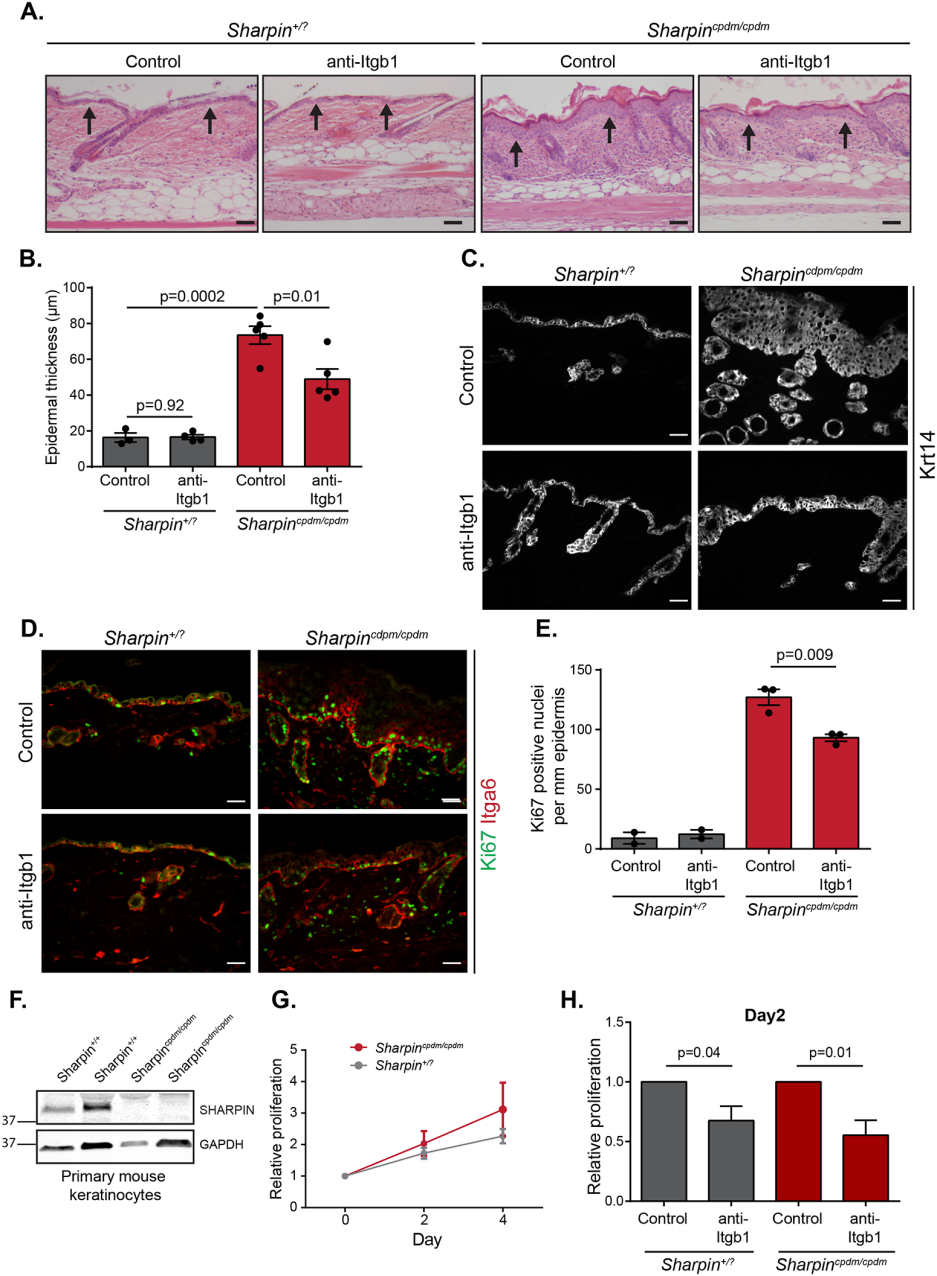
Blocking Itgb1 activity ameliorates the chronic hyperproliferative dermatitis phenotype in *Sharpin*^*cpdm/cpdm*^ mice. (A) Representative H&E-stained skin sections from *Sharpin*^*+/*?^ and *Sharpin*^*cpdm/cpdm*^ mice treated with PBS (control) or anti-Itgb1 (Itgb1 function blocking antibody). Arrows indicate the epidermis. (B) Quantification of epidermal skin thickness from skin sections as depicted in (A) (n = 3 and 4 *Sharpin*^*+/*?^ animals and n = 5 and 5 *Sharpin*^*cpdm/cpdm*^ animals per control and anti-Itgb1, respectively; five individual tissue sections were measured per animal with two measurements per skin section). Data from one out of two experiments with similar results are shown. (C,D) Representative frozen skin sections from *Sharpin*^*+/*?^ and *Sharpin*^*cpdm/cpdm*^ mice treated with PBS (control) or anti-Itgb1, stained for Krt14 (C) or proliferation (Ki67) and basal keratinocytes (Itga6) (D). (E) Quantification of epidermal proliferation from skin sections as depicted in (D) (n = 2 *Sharpin*^*+/*?^ and 3 *Sharpin*^*cpdm/cpdm*^ animals; 4–6 and 19–20 measurements per animal, respectively). (F, G, H) *Sharpin*^*+/*?^ and *Sharpin*^*cpdm/cpdm*^ mouse primary keratinocytes were isolated from 6 weeks old mice and SHARPIN expression (F) and cell proliferation rate (G) were evaluated by western blotting and Cell Proliferation Reagent WST-1, respectively. (H) Relative proliferation of *Sharpin*^*cpdm/cpdm*^ and *Sharpin*^*+/*?^ keratinocytes was quantified after 2 days incubation with 10 μg/ml anti-Itgb1 antibody or isotype IgG control (n = 7 animals per genotype). Scale bars are 50 (A) and 20 μm (C,D). All numerical data are mean ± s.e.m.

Given that SHARPIN indeed inhibits integrin activity in mouse epidermis (Fig 1), we hypothesized that the increased Itgb1 activity might contribute to the *Sharpin*^*cpdm/cpdm*^ dermatitis phenotype. Since epidermal *Itgb1* depletion is lethal [28], *Sharpin*^*cpdm/cpdm*^ and age- and gender-matched control (*Sharpin*^*+/+*^ or *Sharpin*^*+/cpdm*^) mice were systemically treated with an Itgb1 function-blocking antibody or with PBS. *Tnfr1*^*-/-*^ *Sharpin*^*+/*?^ and *Tnfr1*^*-/-*^ *Sharpin*^*cpdm/cpdm*^ mice were not included in this experiment as they display a normal skin phenotype. As expected, the epidermis of *Sharpin*^*cpdm/cpdm*^ mice was much thicker than in control treated mice (Fig 2A–2C). Interestingly, blocking Itgb1 function significantly reduced epidermal thickness in *Sharpin*^*cpdm/cpdm*^, but not in control mice (Fig 2A–2C), without significantly affecting dermal thickness (Panel C in S1 Fig). From each animal the epidermal thickness was measured from five individual tissue sections with two measurements per skin section, thus minimizing the chances of recording experimental outliers. Importantly, plotting all data showed equal variation between measurements and a large difference between experimental conditions (Panel D in S1 Fig).

Staining for the proliferation marker Ki67 indicated that inhibition of Itgb1 in *Sharpin*^*cpdm/cpdm*^ mice reduced the number of proliferating epidermal cells (Fig 2D and 2E), suggesting that Itgb1 inhibition reduces keratinocyte proliferation. To determine if the reduced keratinocyte proliferation is an autonomous effect of Itgb1 inhibition, we cultured *Sharpin*^*cpdm/cpdm*^ and *Sharpin*^*+/*?^ primary mouse keratinocytes, isolated from 6 weeks old mice (Fig 2F and 2G; Panel E in S1 Fig), and evaluated the effect of either Itgb1 function blocking antibody or isotype control antibody on cell proliferation (Fig 2H). While *Sharpin*^*cpdm/cpdm*^ keratinocytes demonstrated comparable or even higher proliferation rate than *Sharpin*^*+/*?^ cells *in vitro* (Fig 2G), integrin inhibition with the Itgb1 function blocking antibody significantly reduced both *Sharpin*^*cpdm/cpdm*^ and *Sharpin*^*+/*?^ primary keratinocyte proliferation (Fig 2H), showing that the Itgb1 blocking antibody inhibits keratinocyte proliferation autonomously and suggesting that amelioration of the chronic proliferative dermatitis (Fig 2A–2C) is, at least partly, linked to decreased keratinocyte proliferation.

### Itgb1 inhibition does not reduce the chronic inflammation in *Sharpin*^*cpdm/cpdm*^ mice

**Fig 3:**
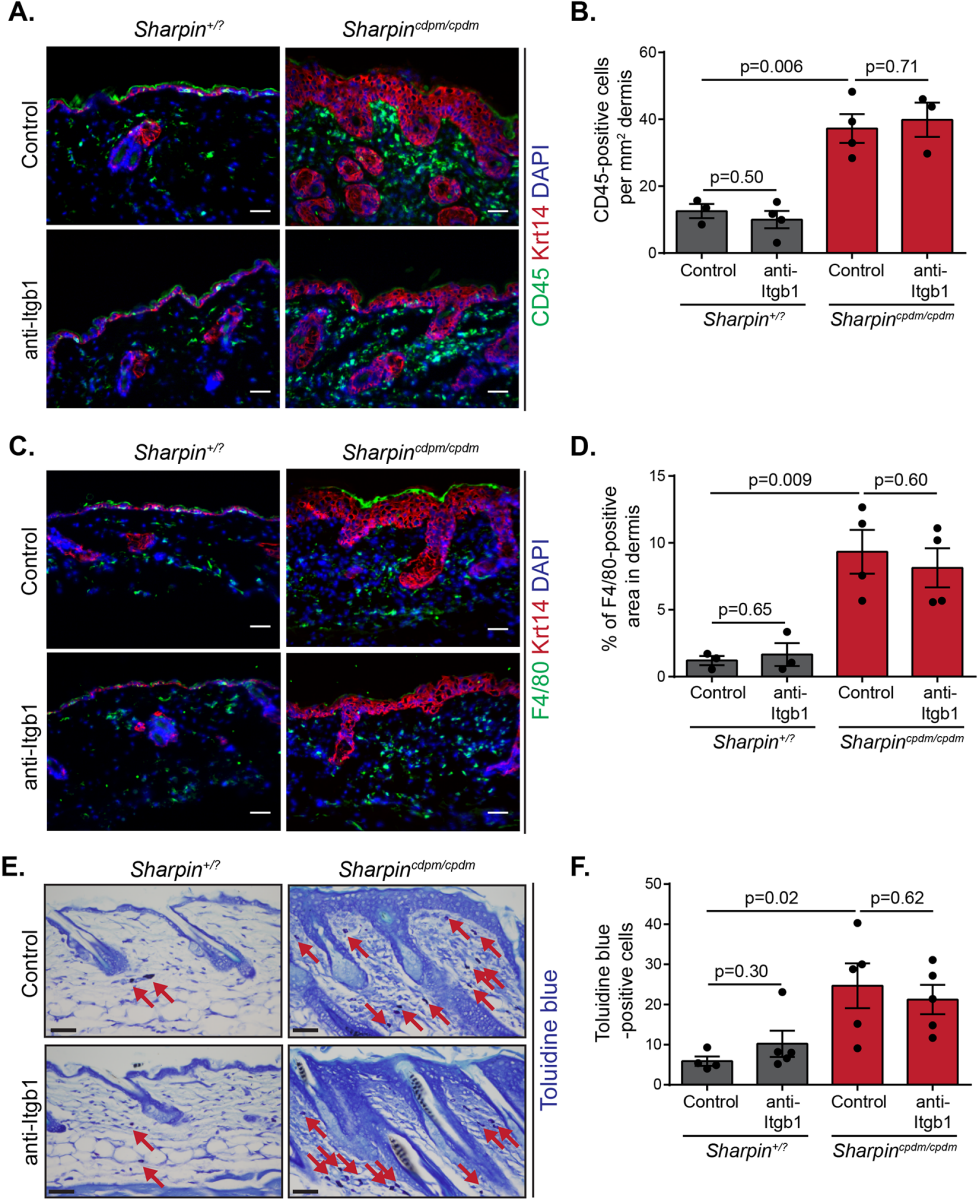
Blocking Itgb1 activity does not inhibit inflammation in *Sharpin*^*cpdm/cpdm*^ mice. (A, C, and E) Representative skin sections from *Sharpin*^*+/*?^ and *Sharpin*^*cpdm/cpdm*^ mice treated with PBS (control) or anti-Itgb1 antibody, and stained for (A) leukocytes (CD45), (C) macrophages (F4/80) and Krt14, or (E) mast cells (Toluidine blue). Nuclei were stained with DAPI (A, C), red arrows indicate toluidine blue positive mast cells (E). Scale bars represent 20 μm (A, C) and 50 μm (E). (B, D, and F) Quantification of (B) dermal leukocyte infiltration (n = 3 or 4 animals; 10–18 measurements per animal), (D) dermal macrophage infiltration (n = 3 or 4 animals; 9–19 measurements per animal) and (F) mast cell infiltration (amount of mast cells/skin section; n = 4 or 5 animals with 10 measurements per animal) from skin sections as depicted in (A, C, and E). Numerical data are mean ± s.e.m.

Chronic inflammation in *Sharpin*^*cpdm/cpdm*^ mice is characterized by enhanced infiltration of immune cells to many tissues including the skin [1,29], as well as increased numbers of white blood cells in the peripheral blood [30]. Interestingly, leukocyte (CD45^+^; Fig 3A and 3B), macrophage (F4/80^+^; Fig 3C and 3D) and mast cell (Toluidine blue^+^; Fig 3E and 3F) infiltration into the dermis of *Sharpin*^*cpdm/cpdm*^ mice was not affected by the Itgb1 function-blocking antibody, indicating that inflammation was not reduced. Also, the increased numbers of white blood cells in the peripheral blood of *Sharpin*^*cpdm/cpdm*^ mice remained elevated or even increased further upon Itgb1 inhibition (Panel A in S2 Fig), further suggesting that the chronic inflammation in *Sharpin*^*cpdm/cpdm*^ mice does not depend on Itgb1.

### Elevated keratinocyte apoptosis in *Sharpin*^*cpdm/cpdm*^ mouse skin is partially rescued by Itgb1 inhibition

**Fig 4:**
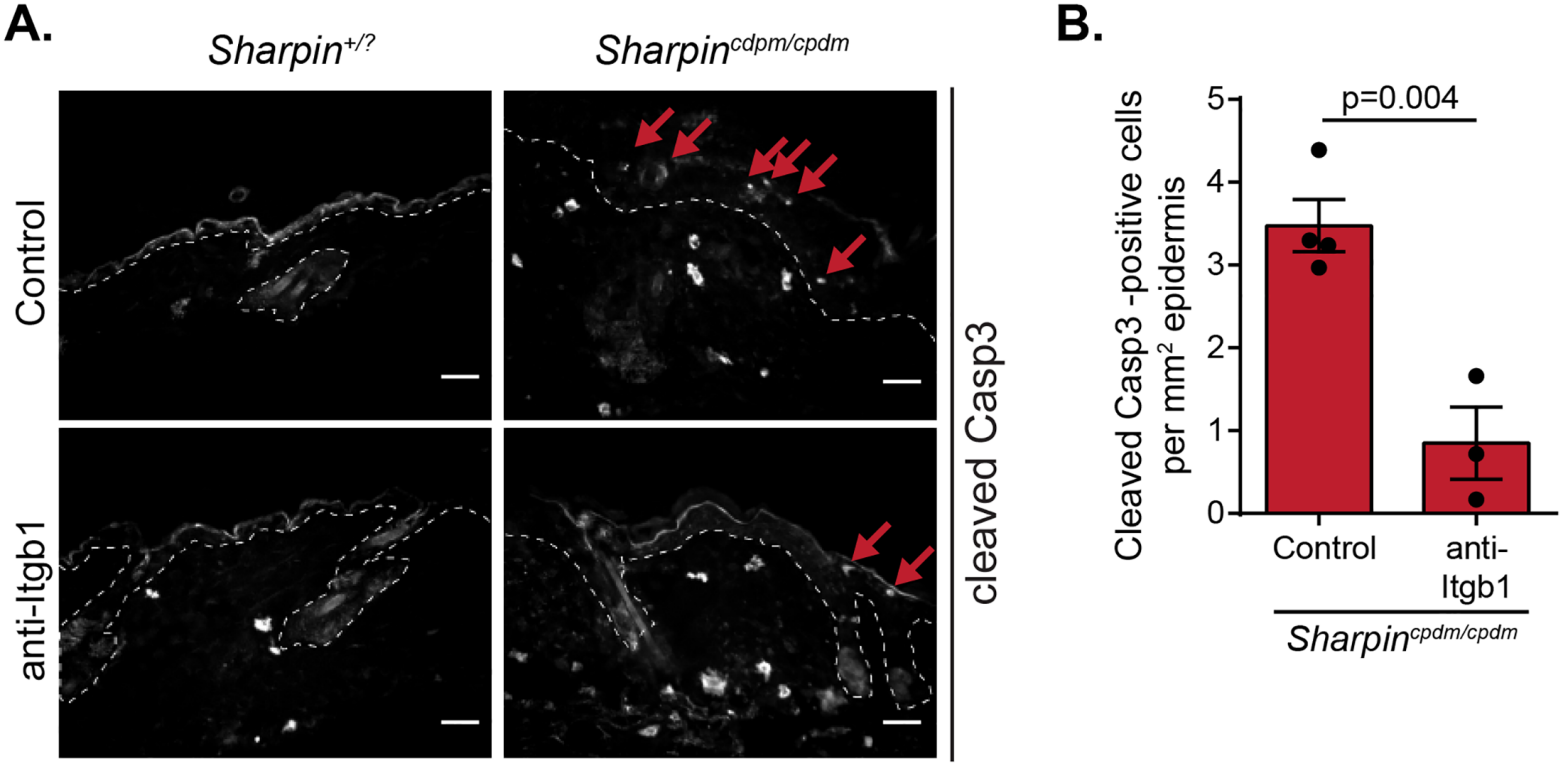
Elevated keratinocyte apoptosis in *Sharpin*^*cpdm/cpdm*^ mouse skin is partially rescued by Itgb1 inhibition. (A) Representative frozen skin sections from *Sharpin*^*+/*?^ and *Sharpin*^*cpdm/cpdm*^ mice treated with PBS (control) or anti-Itgb1, stained for apoptotic cells [cleaved caspase-3 (Casp3)]. Red arrows indicate cleaved caspase-3 positive apoptotic cells. Scale bars represent 20 μm. (B) Quantification of epidermal apoptosis from skin sections as depicted in (A) (n = 4 *Sharpin*^*+/*?^ and 3 *Sharpin*^*cpdm/cpdm*^ animals; 13–18 measurements per animal). Numerical data are mean ± s.e.m.

While the chronic proliferative dermatitis of *Sharpin*^*cpdm/cpdm*^ mice fully depends on TNF-induced keratinocyte apoptosis [6,16,17], Itgb1 antibody treatment did not affect the previously reported [31] elevation of Tnf expression levels in the *Sharpin*^*cpdm/cpdm*^ skin (Panel B and C in S2 Fig), suggesting that altered Tnf expression levels are not mediating the effects of the Itgb1 antibody treatment. However, systemic Itgb1 antibody treatment reduced the increased apoptosis in *Sharpin*^*cpdm/cpdm*^ epidermis (Fig 4A and 4B). Since Tnf levels remained high despite Itgb1 antibody treatment (Panel B and C in S2 Fig), the reduction in apoptotic cell numbers could rather be related to the decreased proliferation as keratinocyte apoptosis balances cell proliferation to control epidermal thickness [32]. These data suggest that inhibition of integrin activity in *Sharpin*^*cpdm/cpdm*^ mice with an anti-Itgb1 antibody ameliorates the chain reaction leading to hyperproliferative dermatitis in the absence of SHARPIN.

## Discussion

The multifunctional adaptor protein SHARPIN regulates integrins [19,20] and TNF-induced NF-kB signaling [4–6], but whether defective integrin regulation also contributes to the *Sharpin*^*cpdm/cpdm*^ skin phenotype has remained unknown [33]. This study now demonstrates that Itgb1 inhibition in *Sharpin*^*cpdm/cpdm*^ mice significantly reduces epidermal proliferation and apoptosis, suggesting a modulatory role for integrins in the chronic proliferative dermatitis phenotype.

Through direct interaction with the integrin cytoplasmic domain SHARPIN inhibits integrin activity, which plays a role in cell adhesion and migration [19,20]. Consistently, increased levels of active Itgb1 were observed in the epidermis of *Sharpin*^*cpdm/cpdm*^ mice [19]. However, as *Sharpin*^*cpdm/cpdm*^ mice suffer from chronic proliferative dermatitis, it has remained unclear whether increased integrin activity in the *Sharpin*^*cpdm/cpdm*^ epidermis is due to lack of SHARPIN or merely a side effect of the dermatitis. Using *Tnfr1*^*-/-*^*Sharpin*^*cpdm/cpdm*^ double knock out mice [6] we now report that Itgb1 activity is also increased in basal *Tnfr1*^*-/-*^ *Sharpin*^*cpdm/cpdm*^ keratinocytes in the absence of proliferative dermatitis, supportive of a direct role for SHARPIN in integrin inhibition *in vivo*. The *Tnfr1*^*-/-*^ *Sharpin*^*cpdm/cpdm*^ mice and other double knock out mice that rescue the *Sharpin*^*cpdm/cpdm*^ inflammatory phenotype [6,16,17] are excellent tools to dissect which *in vivo* functions of SHARPIN are a side effect of the inflammatory phenotype and which phenotypes are independent of the inflammation. In addition, as *Tnfr1*^*-/-*^ *Sharpin*^*cpdm/cpdm*^ mice have a considerably longer life span than *Sharpin*^*cpdm/cpdm*^ mice, which need to be sacrificed by 8 weeks due to severe skin inflammation, these double knock out mice provide means to address whether SHARPIN has important functions later in development and in the development of diseases, such as cancer.

*Sharpin*^*cpdm/cpdm*^ mice display skin thickening (hyperkeratosis), increased keratinocyte proliferation and apoptosis, as well as increased inflammatory cell infiltration [1,2,29]. The phenotype has been shown to originate from defective NF-kB signaling and increased apoptosis induction in response to Tnfr1 activation in keratinocytes, as crosses between *Sharpin*^*cpdm/cpdm*^ mice and *Tnf*^*-/-*^ or *Tnf1r*^*-/-*^ mice fully rescued the chronic proliferative dermatitis [6,16,17]. Transgenic mice with suprabasal expression of Itgb1 displayed similar chronic proliferative dermatitis with deregulated keratinocyte proliferation and skin inflammation, although apoptosis was not significantly affected [24]. In *Sharpin*^*cpdm/cpdm*^ mice, however, the increased Itgb1 activity was primarily observed in basal keratinocytes negative for Keratin-10 or involucrin expression [19]. Our study now shows that inhibition of Itgb1 in *Sharpin*^*cpdm/cpdm*^ mice decreases both keratinocyte proliferation and apoptosis. Importantly, inflammation was not affected, suggesting that the cause of the alleviated phenotype is different from that in the aforementioned *Tnf*^*-/-*^ *Sharpin*^*cpdm/cpdm*^ and *Tnfr1*^*-/-*^ *Sharpin*^*cpdm/cpdm*^ double knockout mice. Thus, while Tnf-induced keratinocyte death drives chronic proliferative dermatitis in *Sharpin*^*cpdm/cpdm*^ mice, increased Itgb1 activity appears to exacerbate the hyperproliferative skin phenotype, consistent with the important role of Itgb1 in keratinocyte proliferation [34].

Recently, SHARPIN was also shown to bind and inhibit the T cell receptor (TCR) in a LUBAC-independent fashion, thereby playing an intrinsic role in the generation of regulatory T cells (Treg cells) [35]. Importantly, transfer of wild type Treg cells into *Sharpin*^*cpdm/cpdm*^ mice considerably alleviated their systemic inflammation [35]. These and our data together show that, though chronic inflammation in *Sharpin*^*cpdm/cpdm*^ mice is triggered by Tnf-mediated apoptosis [6,16,17], other regulatory roles of SHARPIN contribute to different aspects of the complex *Sharpin*^*cpdm/cpdm*^ phenotype.

In summary, our data suggest that Sharpin functions as an integrin inhibitor *in vivo* and that integrins contribute to the chronic proliferative dermatitis phenotype in *Sharpin*^*cpdm/cpdm*^ mice. We therefore postulate that different aspects of the complex *Sharpin*^*cpdm/cpdm*^ phenotype, also in tissues other than skin, could be a result of SHARPIN’s ability to regulate several proteins.

## Materials and methods

### Mice

The C57BL/KaLawRij-*Sharpin*^*cpdm*^/RijSunJ mouse strain (Stock No: 007599 |) with a spontaneous mutation leading to the complete loss of SHARPIN protein [1,2] was acquired from The Jackson Laboratory (Bar Harbor, ME). The colony was maintained in heterozygote breeding and genotyped for the *Sharpin*^*cpdm/cpdm*^ mutation to obtain *Sharpin*^*cpdm/cdpm*^ homozygous mice and littermate control mice (*Sharpin*^*+/+*^ or *Sharpin* ^*+/cpdm*^) for experiments. DNA was extracted with KAPA Mouse Genotyping Kit (KK7302) and the *Sharpin*^*cpdm*^ mutation detected using 40x genotyping assay mix (TaqMan SNP Genotyping Assays, 5793982, Applied Biosystems) and TaqMan Universal PCR Master Mix. *Tnfrsf1a*^*tm1Imx*^ *Sharpin*^*cpdm/cdpm*^ mice [16] (*Tnfrsf1a*^*tm1Imx*^ Stock No: 003242; hereafter called *Tnfr1*^*-/-*^), obtained from Prof. H. Walczak, were also maintained in heterozygote breeding. PCR amplification was used for detection of the deletion in *Tnfr1* gene in somatic DNA. Age-matched mice were used in all the experiments described here. As both male and female *Sharpin*^*cpdm/cdpm*^ mice develop the same symptoms and we have to perform heterozygous breedings, both genders were used in the experiments.

Mice were housed in standard conditions (12-h light/dark cycle) with food and water available *ad libitum*. The viability, clinical signs and behaviour of the mice were monitored daily. For euthanasia, cervical dislocation was used in conjunction with CO_2_. All animal experiments were ethically assessed and authorised by the National Animal Experiment Board and in accordance with The Finnish Act on Animal Experimentation (Animal licence numbers 7522/04.10.03/2012, ESAVI-5588-04.10.07–2014, ESAVI-9339-04.10.07–2016) and The Jackson Laboratory Animal Care and Use Committee (approval number 07005).

### Antibody treatment of mice

Five pairs of 5-week old gender-matched *Sharpin*^*cpdm/cpdm*^ *and Sharpin*^*+/*?^ littermates were injected intraperitoneally with 0.1 ml of 0.05 mg/ml function-blocking anti-mouse ITGB1 antibody (LEAF^™^ purified anti-mouse CD29 Armenian hamster IgG (clone HMB1-1, Biolegend) or PBS twice a week. Mice were injected for a total of 3 weeks (6 injections) and then euthanized by CO_2_ asphyxiation. Complete necropsies were performed as described [36]. Skin was collected in Fekete’s acid-alcohol-formalin. Dorsal skin was also collected in 4% paraformaldehyde for immunofluorescence or fresh frozen for IHC. Blood was collected in EDTA tubes for analysis using the ADVIA 120 Hematology System (Siemens Healthcare Global). Four pairs of 5-week old littermates injected with 0.1 ml of 0.5 mg/ml anti-mouse CD29 antibody or Armenian hamster IgG negative control antibody (Biolegend) showed highly similar results.

### Antibodies

The antibodies used in this study are LEAF^™^ purified Anti-mouse CD29 Armenian hamster IgG (Biolegend; HMB1-1), Armenian hamster IgG negative control (Biolegend), Itga6 (Serotec; NKI-GoH3; 1:200 in immunohistochemistry of frozen sections (IHC-Fr), cleaved Casp3 (Cell Signaling; 5A1E; 1:400 IHC-Fr), Ki67 (Abcam; ab66155; 1:300 IHC-Fr), CD45 (PTPRC) (BD Pharmingen; 30-F11; 1:250 in IHC-Fr, 1:200 in FACS), F4/80 (CiteAb; MF48020; 1:50 in IHC-Fr), Krt14 (Biosite; PRB-155P; 1:600 in IHC-Fr), Tnf (Abcam; ab6671; 1:200 in IHC-Fr), CD31 (PECAM1) (Biolegend; 1:50 in FACS), active Itgb1 (BD Pharmingen; 9EG7; 1:100 in IHC-Fr, 1:50 in FACS), total Itgb1 (CD29-Alexa Fluor 647) (Biolegend; HMβ1–1; 1:50 in FACS), CD49f (Itga6) (Biolegend; GoH3; 1:20 in FACS), SHARPIN (Proteintech; 1:1000 WB), Krt14 (Covance; 1:1000 WB), Vimentin (D21H3, Cell Signalling; 1:1000 WB), GAPDH (5G4 Mab 6C5, Hytest; 1:1000 WB), DyLight 680- or 800-conjugated IgGs (Thermo Scientific; 1:5000 WB) and AlexaFluor 488/568/647-conjugated secondary Igs (Invitrogen; 1:300 in IHC-Fr and FACS).

### Immunofluorescence

O.C.T. (Sakura) embedded frozen skin sections were fixed (4% paraformaldehyde), quenched (100mM glycine in PBS), and blocked/permeabilized (2% BSA, 0.1% Triton X100 in PBS). Samples were labeled with primary antibodies (1 hour at RT in 2% BSA in PBS), washed three times and labeled with fluorescent secondary antibodies. After washes, samples were mounted in Vectashield mounting medium (Vector Laboratories) and viewed using a 20x objective on either a 3i Marianas Spinning disk confocal microscope (Intelligent Imaging Innovations) or Zeiss Axiovert 200M with Yokogawa CSU22 spinning disk confocal microscope unit with Hamamatsu Orca ER CCD camera (Hamamatsu Photonics K.K.). Images were processed and quantified using Fiji image analysis software [37].

### Isolation and culture of mouse primary keratinocytes

6–8 weeks old littermate mice were sacrificed, dorsal skin was shaved and a piece of dorsal skin (1.5 cm x 1.5 cm) was harvested in cold PBS. Tissue was cut further to smaller pieces, rinsed with Hank’s Balanced Salt Solution without Ca^2+^ and Mg^2+^ (HBSS; Sigma), and incubated overnight in 0,25% porcine trypsin (Sigma) in HBSS at +4°C on a shaker. Next day, the dermis was removed with forceps, the remaining epidermis was chopped with a scalpel and incubated in 0.2% Collagenase XI (Sigma) at 37°C incubator for 30 min. Keratinocytes were dissociated from the tissue by pipetting the suspension every 5–10 minutes. Keratinocytes were filtered through a cell strainer (70 μm; BD Biosciences). Finally, cells were incubated with 20 U/ml DNase I (Roche) for 5 min on ice and spun down.

Primary keratinocytes were cultured on a 6-well plate coated with 20 μg/ml Collagen Type I from rat tail (Merck Millipore) in FAD medium [Dulbecco’s Modified Eagle’s Medium:Ham’s F12 Nutrient Mixture (GIBCO) 3.5:1.1] supplemented with 10% fetal bovine serum (FBS) (chelated using Bio-Rad Chelex^®^ 100 Chelating Resin), 5 μg/ml insulin, 100 pM choleratoxin, 10 ng/ml epidermal growth factor (EGF), 100 U/ml sodium pyruvate, 100 μg/ml penicillin-streptomycin and 0.5 μg/ml hydrocortisone (all from Sigma Aldrich if not otherwise mentioned) in 5% CO_2_ and 32°C.

### Keratinocyte proliferation assay

Equal amounts of primary mouse keratinocytes were plated on 96-well plates coated with 20 μg/ml rat tail Collagen Type I. One day after seeding, the keratinocytes were treated with 10 μg/ml anti-Itgb1(Anti-mouse CD29 Armenian hamster IgG) or isotype IgG control antibody. Theamount of cells) in each well was quantified with Cell Proliferation Reagent WST-1 (Roche, Sigma Aldrich) according to the manufacturer’s instructions and a BioTek Synergy H1 Hybrid Multi-Mode Microplate Reader. The relative proliferation was calculated by normalizing the amount of cells in each time point to day zero (Fig 2G) or by subtracting the amount of cells at day zero from the value at day two (corrected cell amount), followed by normalizing to the corrected cell amount of IgG control. A minimum of two wells per keratinocyte primary cell line were analysed per time point.

### Flow cytometry

The active conformation specific Itgb1 antibody (clone 9EG7, BD Pharmingen) was concentrated with an Amicon^®^ Ultra filter centrifugation and directly conjugated to Alexa Fluor 647 with APEX^®^ antibody labelling kit (Thermo Fischer Scientific). Cells were fixed with 2% paraformaldehyde in PBS for 10 min at RT, after which they were centrifuged and washed with cold Tyrodes buffer (10 mM Hepes-NaOH pH 7.5, 137 mM NaCl, 2.68 mM KCl, 0.42 mM NaH_2_PO_4_, 1.7 mM MgCl_2_, 11.9 mM NaHCO_3_, 5 mM glucose). The cells were stained with directly fluorochrome-conjugated antibodies [CD31-Pacific Blue (PECAM1), CD45-Pacific Blue (PTPRC), CD49f-488 (Itga6, Clone: GoH3) (all from BioLegend), and CD29-AlexaFluor 647 or active Itgb1 (clone 9EG7) -647] in 100 μl Tyrodes for 30 min RT, washed with Tyrodes and resuspended in PBS. Filtered cells were analysed using BD LSRFortessa (BD Biosciences) and FlowJo analysis software (Tree Star).

### Western blotting

Protein content in primary mouse keratinocytes was analysed by immunoblotting using standard western blotting techniques and the Odyssey LICOR imaging system.

### Statistical analysis

All statistical analyses were performed using GraphPad Prism (GraphPad Software, San Diego, USA). Normal distribution was assumed for the data and unpaired Student's t-test was used for all experiments. A p < 0.05 was considered significant.

## Supporting information

S1 Fig Experiments in (C-E) show data from *Sharpin*^*+/*?^ and *Sharpin*^*cpdm/cpdm*^ mice treated with PBS (control) or anti-Itgb1 (Itgb1 function blocking antibody). (A,B) FACS analysis (A) and quantification (B) of active Itgb1 (10 μg/ml Rat Anti-Mouse CD29, Clone 9EG7) labelling specificity compared to isotype IgG control (10 μg/ml Rat IgG2a, κ isotype control) in primary mouse keratinocytes using AlexaFluor647 conjugated anti-rat secondary antibody. Samples were analyzed by flow cytometry at SSC/FSC and APC-A channels, and background fluorescence was subtracted before normalization to Isotype IgG (mean +/-SEM; n = 4 mice). (C) Quantification of dermal skin thickness from skin sections similar to those depicted in Fig 2A (n = 4 or 5 animals with 10 measurements per animal). (D) Dot plot of all individual measurements of epidermal skin thickness from skin sections similar to those depicted in Fig 2A (five individual tissue sections were measured per animal with two measurements per skin section). The plot in Fig 2B shows averages per animal. (E) Krt-14 (marker for keratinocytes), vimentin (marker for fibroblasts) and GAPDH expression levels in keratinocytes isolated from *Sharpin*^*+/+*^, *Sharpin*^*+/-*^ and *Sharpin*^*cpdm/cpdm*^ mice, as well as mouse embryonic fibroblast (MEF). Keratinocyte isolation purity was evaluated by measuring the Krt14/vimentin ratio, normalized to GAPDH, with MEFs serving as a control fibroblast cell line. Molecular weight markers are indicated (KDa). All numerical data are mean ± s.e.m. Scale bars represent 20 μm.
(TIF)

S2 Fig Blocking Itgb1 activity does not affect inflammation or Tnf levels in *Sharpin*^*cpdm/cpdm*^ mice. Experiments show data from *Sharpin*^*+/*?^ and *Sharpin*^*cpdm/cpdm*^ mice treated with PBS (control) or anti-Itgb1 (Itgb1 function blocking antibody). (A) Quantification of immune cell populations from peripheral blood. WBC, white blood cells; LUC, large unstained cells. (B,C) Representative skin sections stained for Tnf and nuclei (DAPI) (B) and quantification of dermal Tnf levels (C) (n = 2 and 4 *Sharpin*^*+/*?^ animals in control and anti-CD29 group, respectively, and 5 in each *Sharpin*^*cpdm/cpdm*^ group, 10–40 measurements per animal). Dotted line marks the basement membrane. All numerical data are mean ± s.e.m. Scale bars represent 20 μm.
(TIF)
