# Supplementary figures and images for: Integrin beta 1 inhibition alleviates the chronic hyperproliferative dermatitis phenotype of SHARPIN-deficient mice
Source: PLoS One. 2017 Oct 17;12(10):e0186628. doi: 10.1371/journal.pone.0186628 (PMC5645136; doi:10.1371/journal.pone.0186628)

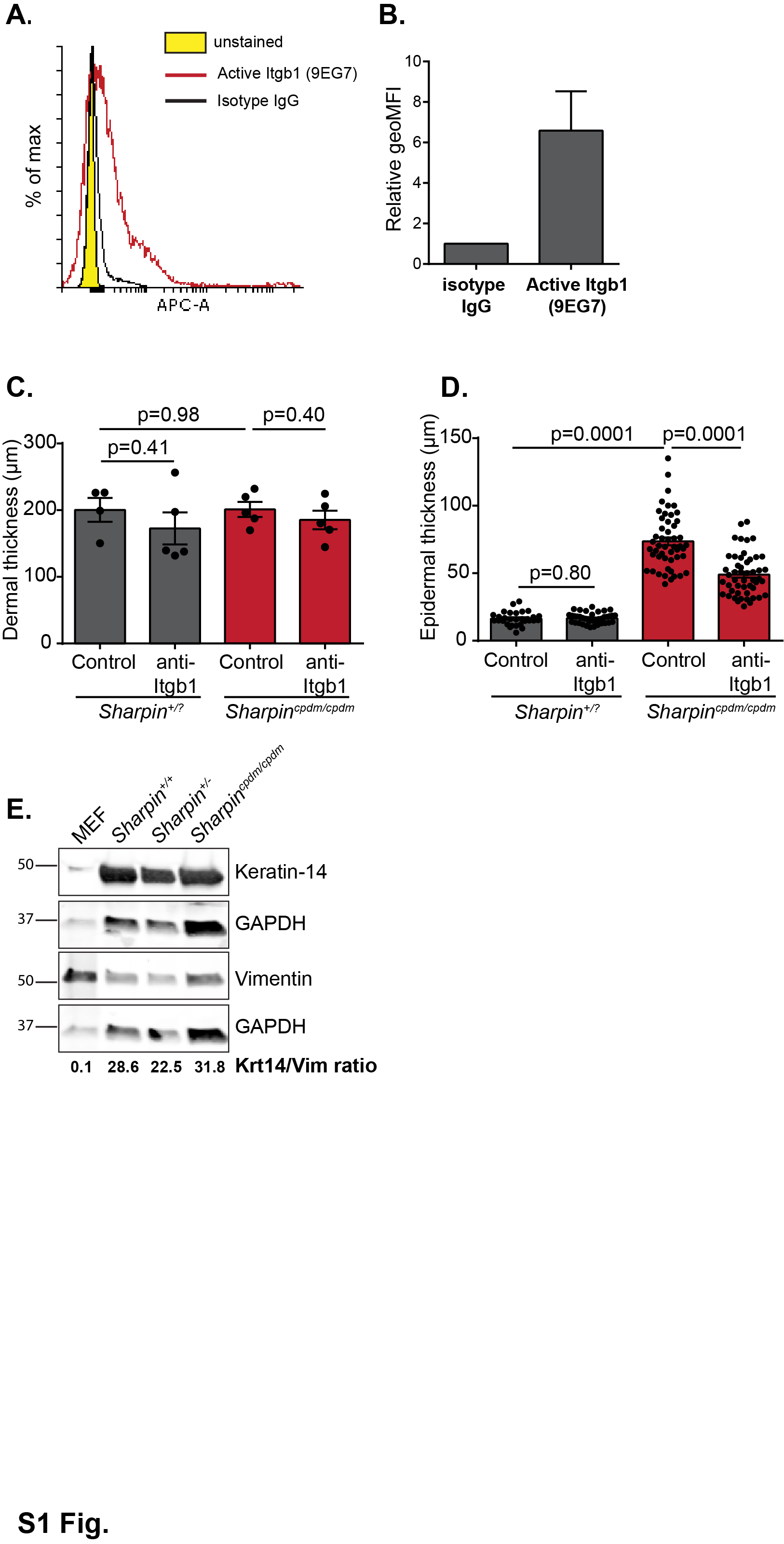

Supplement: S1 Fig — (A,B) FACS analysis (A) and quantification (B) of active Itgb1 (10 μg/ml Rat Anti-Mouse CD29, Clone 9EG7) labelling specificity compared to isotype IgG control (10 μg/ml Rat IgG2a, κ isotype control) in primary mouse keratinocytes using AlexaFluor647 conjugated anti-rat secondary antibody. Samples were analyzed by flow cytometry at SSC/FSC and APC-A channels, and background fluorescence was subtracted before normalization to Isotype IgG (mean +/-SEM; n = 4 mice). (C) Quantification of dermal skin thickness from skin sections similar to those depicted in Fig 2A (n = 4 or 5 animals with 10 measurements per animal). (D) Dot plot of all individual measurements of epidermal skin thickness from skin sections similar to those depicted in Fig 2A (five individual tissue sections were measured per animal with two measurements per skin section). The plot in Fig 2B shows averages per animal. (E) Krt-14 (marker for keratinocytes), vimentin (marker for fibroblasts) and GAPDH expression levels in keratinocytes isolated from Sharpin+/+, Sharpin+/- and Sharpincpdm/cpdm mice, as well as mouse embryonic fibroblast (MEF). Keratinocyte isolation purity was evaluated by measuring the Krt14/vimentin ratio, normalized to GAPDH, with MEFs serving as a control fibroblast cell line. Molecular weight markers are indicated (KDa). All numerical data are mean ± s.e.m. Scale bars represent 20 μm. (TIF) [file pone.0186628.s001.tif]

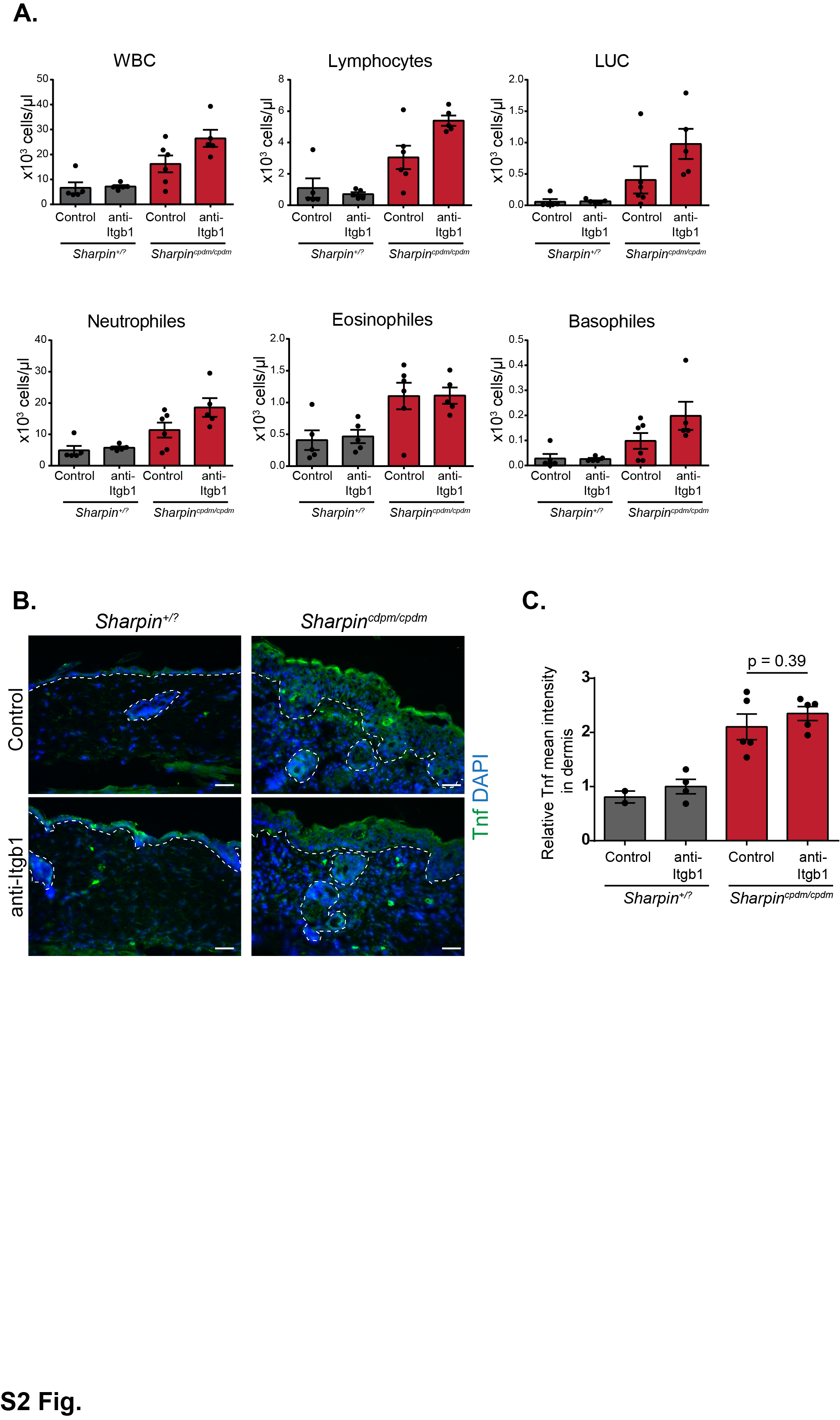

Supplement: S2 Fig — Experiments show data from Sharpin+/? and Sharpincpdm/cpdm mice treated with PBS (control) or anti-Itgb1 (Itgb1 function blocking antibody). (A) Quantification of immune cell populations from peripheral blood. WBC, white blood cells; LUC, large unstained cells. (B,C) Representative skin sections stained for Tnf and nuclei (DAPI) (B) and quantification of dermal Tnf levels (C) (n = 2 and 4 Sharpin+/? animals in control and anti-CD29 group, respectively, and 5 in each Sharpincpdm/cpdm group, 10–40 measurements per animal). Dotted line marks the basement membrane. All numerical data are mean ± s.e.m. Scale bars represent 20 μm. (TIF) [file pone.0186628.s002.tif]
